# Protective Isolation for Patients with Haematological Malignancies: A Pilot Study Investigating Patients’ Distress and Use of Time

**Published:** 2017-10-01

**Authors:** O. Annibali, C. Pensieri, V. Tomarchio, V. Biagioli, M. Pennacchini, A. Tendas, V. Tambone, M.C. Tirindelli

**Affiliations:** 1 Unit of Hematology, Stem Cell Transplantation, Transfusion Medicine and Cellular Therapy, Campus Bio-Medico University of Rome, Rome, Italy; 2 Institute of Philosophy of Scientific and Technological Activity, Campus Bio-Medico University of Rome, Rome, Italy; 3 Department of Biomedicine and Prevention, School of Nursing, University of Rome Tor Vergata, Rome, Italy; 4 Hematology Unit, Ospedale Sant’Eugenio Hospital, Roma, Italy

**Keywords:** Patient isolation, Social isolation, Hematopoietic stem cell transplantation, Time perception

## Abstract

**Background:** Patients with haematological malignancies are often hospitalized in protective isolation until full neutrophil recovery in order to prevent infections. This descriptive pilot study evaluate the level of isolation-related distress and the use of free time in a sample of Italian onco-haematological patients who were hospitalized in protective isolation.

**Materials and Methods:** Participants were 18 patients hospitalized in hematologic ward to receive induction therapy (n=12) or autologous stem cell transplant (n=6). They completed a self-report questionnaire before discharge.

**Results: **Participants reported a moderate level of isolation-related distress, anxiety, and boredom: the more the anxiety and the boredom, the more the distress (r=.77; P<.001), (r=.79; P<.001), respectively. The activities performed during isolation were: watching TV (72.2%), reading (55.6%), thinking (33.3%), surfing in Internet or using PC (33.3%), and playing games or making cross-words (16.7%). Participants who reported pessimistic thinking had higher isolation-related distress (P=.004) as well as anxiety (P<.001) and boredom (P=.001).

**Conclusion: **Haematology Units should support isolated patients in spending their time in recreational activities, allowing more contacts with immediate relatives and friends, providing free TV and Wi-Fi connection inside the room. In addition, patients should have to keep themselves physically active. Isolation-related distress could also be reduced by providing psychological support.

## Introduction

 Patients with haematological malignancies, especially those who undergo haematopoietic stem cell transplantation (HSCT), are often hospitalized in protective isolation until full neutrophil recovery in order to prevent infections^1^^-^^2^. Protective isolation measures are not supported by definitive scientific evidence and every single center acts in different ways. In Italy, protective strict isolation is commonly used for patients receiving high-dose chemotherapy or for those receiving autologous HSCT. Usually, the patient is alone in a germ-free room during the entire hospitalization period, he has to follow preventive self-care behaviors and he cannot receive visits except in accordance with ward policy.

During isolation, patients have to deal not only with treatment-related complications and adverse events such as physical symptoms and changes in body image but also with isolation-related psychological distress, including loss of control and lack of contact with family members. Although, some patients are able to adapt themselves to this new condition, usually being alone in a hospital room for a certain period of time could become a devastating experience. In some cases, patients can suffer from loneliness, depression, anxiety, confusion, feelings of esclusion, which in some case could lead to violence, escape behavior or suicidal ideation. As a consequence, it is necessary to investigate deeply the level of isolation-related distress and the associated variables. In particular, patients who spend their time in recreational activities, such as the use of technology, may better cope with isolation experience.

The aim of this study was to evaluate the level of isolation-related distress and to analyze the way in which isolated patients spend their time during the admission into hospital.

## MATERIALS AND METHODS

**Design**

From August 2010 to May 2012, we conducted a descriptive prospective pilot study aimed to evaluate the level of isolation-related distress and the use of free time during the admission into hospital in a cohort of patients with haematological malignancies hospitalized in the Haematology ward at Campus Bio-Medico University Hospital in Rome. The study was approved by the Ethics Committee of Campus Bio-Medico University.

**Setting**

The hematology ward where the study was conducted included 4 HEPA-filtered single rooms with en suited toilet where patients remained until discharge^3^^-^^9^. Patients could not leave the room, unless required, for example in case of medical procedures (e.g. radiologic) or emergencies. The policy for visitors allowed only one family member/friend visits twice a day for two-hours in the morning (12 p.m.–2 p.m.) and in the afternoon (6–8 p.m.). Dietary restrictions and rigorous preventive measures, such as shower and mouth care, were mandatory. 

**Participants and data collection**

Participants were patients affected by haematologic malignancies who were hospitalized in protective isolation in order to receive chemotherapy followed or not by autologous HSCT. Patients were invited to participate in the study by the clinical staff of the Unit. After having signed an informed consent, participants were asked to complete a self-report questionnaire at the end of their hospitalization. The questionnaire included data about socio-demographic characteristics, relational network and visitors, use of time during hospitalization and use of technology. In addition, their level of isolation-related distress, like discomfort, lack of happiness, boredom, lack of serenity, anxiety, and anger was measured with 6 items on a 5-point Likert scale; higher scores indicated higher isolation-related distress. Boredom and anxiety were also assessed with single items ranging from 0 to 10.

Descriptive statistics were analyzed for each variables studied. The levels of distress, anxiety, and boredom were analyzed in relation to the use of time through univariate analysis of variance (ANOVA).

## Results

 Eighteen patients were enrolled in this study, 12/18 received standard dose chemotherapy, followed in n=6/18 by autologous HSCT. Table 1 shows the socio-demographic and clinical characteristics of the sample. Median age was 55 years (21- 72). 9/18 patients were male (50%) and 9/18 were female (50%). The duration of hospitalization varied from 17 to 122 days. Participants reported to have from 2 to 9 close relatives. The majority of patients (n=13/18; 72.2%) received visits from relatives every day during their hospital period, while 4/18 (22.2%) patients received 2-3 visits per week, and 1/18 (5.6%) patient received only one visit per week. Only 6/18 (33%) reported visits from friends or not closed relatives. 

**Table 1:** Demographic and clinical characteristics of the 18 participants

**Characteristic**	**N**	**%**
Age Median (range)	55 (21-72)	-
Sex		
Male	9	50.0
Female	9	50.0
Marital status		
Single	3	16.7
Married	13	72.2
Divorced	1	5.6
Widow	1	5.6
Educational level		
Primary school	1	5.6
Secondary school	9	50.0
High school	7	38.9
University	1	5.6
Work status		
Employed	6	33.3
Unemployed	12	66.7
Diagnosis		
Acute leukemia	10	55.6
Non-Hodgkin Lymphoma	5	27.8
Multiple Myeloma	3	16.7
Status of the disease		
Newly diagnosed	4	22.2
Partial response	2	11.1
Relapsed	4	22.2
Complete remission	8	44.4
Number of previous admissions		
1st admission	9	50.0
2nd or more admission	9	50.0
Days of hospitalization Median (range)	49 (17-122)	-

Participants reported a mean level of isolation-related distress of 2.94 (SD=1.02), indicating moderate distress. Their level of anxiety, as measured through a single item, was moderate as well (mean=5.22; SD=3.67). Their level of boredom, as measured through a single item, was also moderate (mean=5.94; SD=3.24). These variables were positively associated with each other: the more the anxiety and the boredom, the more the distress (r=.77; P<.001), (r=.79; P<.001), respectively. 

 In terms of frequency of each isolation-related feeling (Table 2), the majority of patients reported never (38.9%) or rarely (22.2%) discomfort. The most experienced feeling was boredom (61.1%) and several patients complained of being always (11.1%) or often (50%) bored. Most of the patients reported to be rarely (33.3%) or never (44.4%) happy, but nearly half of the sample (44.4%) felt often (33.3%) or always (11.1%) calm. Only few patients experienced anger (27.8%); the majority of them (67.7%) rarely (16.7%) or never (50%) got angry. While 8/18 (44.5%) patients were always (16.7%) or often (27.8%) anxious, 10/18 (55.5%) experienced anxiety rarely (22.2%) or never (33.3%). 

**Table 2:** Frequencies and percentages of feelings reported during isolation

	**Always ** n (%)	**Often ** n (%)	**Sometimes** n (%)	**Rarely** n (%)	**Never ** n (%)
**Discomfort**	1 (5.6)	6 (33.3)	-	4 (22.2)	**7 (38.9)**
**Boredom**	2 (11.1)	**9 (50.0)**	-	3 (16.7)	4 (22.2)
**Anxiety**	3 (16.7)	5 (27.8)	-	4 (22.2)	**6 (33.3)**
**Anger**	4 (22.2)	1 (5.6)	1 (5.6)	3 (16.7)	**9 (50.0)**
**Happiness**	1 (5.6)	1 (5.6)	2 (11.1)	6 (33.3)	**8 (44.4)**
**Serenity**	2 (11.1)	**6 (33.3)**	2 (11.1)	**6 (33.3)**	2 (11.1)

Note: in bold the highest frequency for each feeling

Regarding the perception and use of time during isolation, only few patients reported that they spent their time in sleeping (n=2/18; 11.1%) or resting (n=3/18; 16.7%). Many patients answered they had something to do (n=7/18; 38.9%). Unfortunately, half of the sample (n=9/18; 50%) reported pessimistic thinking about their future and their health. On the other hand, many patients (n=11/18; 61.1%) reported that they spent their time thinking about their lives and their future goals during isolation. Besides, many patients (n=10; 55.6%) reported the desire of staying with other people. 

Participants who reported pessimistic thinking had higher isolation-related distress (P=.004), as well as anxiety (P<.001) and boredom (P=.001). In addition, patients who had something to do during isolation felt less anxiety than those who had nothing to do (P=.006).

Since many patients (n=11/18; 61.1%) used Internet in their daily life for checking emails, surfing social networking sites or reading e-books, nearly half of the sample (n=8; 44.4%) expressed a desire to have the Wi-Fi connection inside their hospital rooms. Although most of the patients practiced at least a sport in their daily life (n=12/18; 66.7%), they spent their time in sedentary activities during the hospitalization period. The most frequent activities included: watching TV (n=13/18; 72.2%), reading (n=10/18; 55.6%), thinking (n=6/18; 33.3%), surfing the Internet or using the PC (n=6; 33.3%), and playing games or making cross-words (n=3/18; 16.7%).

Most of the participants believed to have been on isolation for a lot (n=9/18; 50%) or too much time (n=4/18; 22.2%), while only few of them believed to have spent enough (n=3/18; 16.7%) or short (n=2/18; 11.1%) time in isolation. 

## Discussion

 This prospective descriptive pilot study assessed the level of isolation-related distress and the use of time during hospitalization in 18 patients with haematologic malignancies hospitalized in a University Hospital in Italy. Isolation’s duration was comparable with other haematology centers in Italy, and was perceived as long/too long by most of the sample. This could suggest the need of an early discharge after high-dose chemotherapy and the possibility to complete the medical care at home.

About the use of time during isolation, findings emphasized the importance of having a TV and a free Wi-Fi connection inside the room; as a consequence, all haematology centers should provide isolation rooms with these services. However, even when patients can spend their time watching TV, reading, thinking and surfing Internet or using the PC, they might experience boredom. In addition, the majority of patients experienced boredom as well as anxiety, and suffered from isolation-related distress. Boredom, defined as the dissatisfaction associated with the perception of time as passing slowly, has been described as an attribute of environmental loneliness .This underlines the importance for isolated patients to manage their time alone in order to avoid experiencing boredom, which can make them feel lonelier^11^^-^^27^. 

Moreover, participants reported their desire of staying with other people, which can result as a crucial aspect to fight against boredom and anxiety. Spending more time with family members/friends might improve patients’ quality of life during isolation. For example, haematology centers could prolong visiting hours, install an intercom, or create common protective spaces for family meeting. Other efforts to reduce isolation discomfort could include the use of Tablet to enhance internet’s relationship with family members, friends and patients’ communities or to invest free time in games, films, musics and also the creation of virtual world like 3D immersive games or 3D simulated environment in which participants could live in virtual reality. Recently, some medical fields like rehabilitation medicine have used this type of technological support with success^28^. It is important to improve patient’s physical status and at the same time to support psychological aspect that would influence the patient’s well-being such as emotional distress and lack of social support^29^.

In addition, since many patients have already incorporated an exercise program into their daily life, and during isolation they may experience loss of muscles and weight due to physical complications, they should have to keep themselves physically active to help maintain muscle strength and a healthy weight^30^^-^^31^. Meanwhile, we could improve the physical activity of patients through the help of technology: patients could use workout and fitness games on video game consoles via Tablets or Smartphones to improve motion. These forms of technology have been successful in providing an opportunity for patients to deal with isolation and boredom and also in improvement of outcome following stroke rehabilitation^32^^,^^33^. 

Patients might also benefit from psychological support as half of the participants with increased isolation-related distress engaged in pessimistic thinking about their future and their health. 

## CONCLUSION

 In conclusion, it is essential to ensure that the best treatment will be given to a patient, but there is “*no profit in curing the body if in the process you destroy the soul*” (Samuel H. Golter), so every single Hematology Unit in Hospitals should support isolated patients by allowing them to spend more time with relatives and friends. Scientific data demonstrated that personal contact is one of the best forces to cope with distress and isolation. Each ward should provide patients with recreational opportunities, including free TV and Wi-Fi connection inside the room. Furthermore, patients should keep themselves physically active. Isolation-related distress could also be reduced by providing psychological support. 
